# Exploration of treatment‐free remission in CML, based on molecular monitoring

**DOI:** 10.1002/cam4.6849

**Published:** 2023-12-22

**Authors:** Zhao Zhang, Xianghui Zhou, Xin Zhou, Zhipeng Cheng, Yu Hu

**Affiliations:** ^1^ Wuhan Union Hospital Wuhan Hubei China

**Keywords:** BCR::ABL1, chronic myelogenous leukemia, deep molecular response, molecular monitoring, treatment‐free remission, tyrosine kinase inhibitor discontinuation

## Abstract

**Background:**

Typical chronic myelogenous leukemia (CML) is a myeloproliferative neoplasm caused by t(9; 22)(q34; q11) translocation. This chromosomal translocation forms the BCR::ABL1 fusion gene. The tyrosine kinase encoded by the BCR::ABL1 is considered to be the main pathogenic diver. BCR::ABL1 is not only a therapeutic target, but also a monitoring target. Monitoring of BCR::ABL1 reveals the progression of the disease and guides the next treatment. Now for CML, the target of treatment has been focused on treatment‐free remission (TFR).

**Methods:**

We conducted a literature review of current developments of treatment‐free remission and molecular monitoring methods.

**Results:**

More effective and sensitive CML monitoring methods such as digital droplet PCR (ddPCR) and next generation sequencing (NGS) have further studied the measurable residual disease (MRD) and clonal heterogeneity, which provides strong support for the exploration of TFR. We discussed some of the factors that may be related to TFR outcomes at the molecular level, along with some monitoring strategies.

**Conclusion:**

Currently, predictive indicators for treatment‐free remission outcomes and recurrence are lacking in clinical practice. In future, treatment‐free remission research should focus on combining the clinical indicators with molecular monitoring and biological markers to personalize patient conditions and guide clinicians to develop individualized treatment plans, so that more patients with CML can achieve safer and stabler treatment‐free remission.

## Graphical abstract

This study aimed to review the factors that may be related to achieving treatment‐free remission (TFR) in patients with chronic myeloid leukemia (CML), at the molecular level, and discussed some monitoring strategies, based on the currently available clinical guidelines and outcomes of some clinical trials.

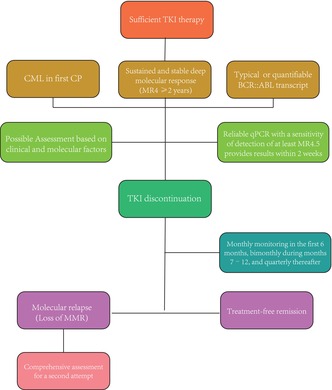

## 1 INTRODUCTION

Chronic myeloid leukemia (CML) is a disease characterized by the clonal proliferation of pluripotent stem cells that undergo malignant transformation resulting in a significant increase in mature and immature granulocytes. Typical CML begins with the t(9;22)(q34;q11) translocation, which can cause the fusion of the proto‐oncogene ABL fragment on chromosome 9 with the BCR gene fragment on chromosome 22,^1^ resulting in the fusion gene, BCR::ABL1, which encodes a large number of abnormal tyrosine kinases that promote disease development. ABL overexpression is a key therapeutic target for CML. Since the emergence of tyrosine kinase inhibitors (TKIs) in 2001, prognosis of patients with CML has improved greatly.^2^ This progress has improved the life expectancy of patients with CML to as much as that of the normal population.^3^ Several TKIs have been used clinically till date. Long‐term use of imatinib as a first‐line treatment for patients with primary CML‐chronic phase confirmed the 10‐year survival rate to be 80%–90%. Introduction of second‐generation TKIs (such as nilotinib, dasatinib, bosutinib, and radotinib) and the third‐generation TKI (such as ponatinib) further accelerated and improved the treatment response rates and response depths in patients.^4^ A subset of studies confirmed that patients receiving second‐generation TKIs have higher and faster major molecular response (MMR) rates than those receiving imatinib.^5^, ^6^ Third‐generation TKIs have shown stronger therapeutic efficacy and effectiveness in some patients with drug resistance mutations.^7^, ^8^ The formulation of different treatment plans must consider various factors, including patient complications, disease progression, TKI resistance, drug toxicity, and treatment costs. In the past, in pursuit of higher overall survival (OS) rates, CML treatment was often lifelong. However, lifelong use of TKI might cause adverse events,^9^ which could affect the patient's physical condition and quality of life, leading to a decline in patient compliance, and affecting the final treatment response.^10^ Therefore, in recent years, the ultimate therapeutic goal for CML has focused on treatment‐free remission (TFR), that is, stopping TKI therapy after achieving a sustained deep molecular response (DMR, Table 1) without affecting the expected therapeutic effect. The realization of TFR can reduce drug‐induced adverse events, lessen the financial burden on patients, improve their quality of life, and be important in some special circumstances, such as pregnancy. Sustained DMR is a prerequisite for achieving TFR; therefore, continuous and precise molecular monitoring is essential. Monitoring methods for BCR::ABL1 have evolved from the initial reverse transcription‐PCR (RT‐PCR) to quantitative RT‐PCR (qRT‐PCR) and, more recently, to digital PCR.^11^ ABL kinase region mutations can be analyzed by direct sequencing (Sanger sequencing with sensitivity of 10%–20%) or next‐generation sequencing (NGS) of the BCR::ABL kinase region to identify the ABL kinase region point mutations^12^ and TKI resistance, and guide subsequent treatment selection. Although TFR is still in the exploratory stage, the emergence of new monitoring methods and high‐quality clinical research (Tables 2 and 3) can make its widespread realization possible.

**TABLE 1:** Milestones in CML treatment.

Milestones		Log reduction	BCR::ABL% (IS)
EMR	McyR	−1	10%
	CCyR	−2	1%
MMR	MR3	−3	0.1%
	MR4	−4	0.01%
DMR	MR4.5	−4.5	0.0032%
	MR5	−5	0.001%

Abbreviations: CCyR, complete cytogenetic response; DMR, deep molecular response; EMR, early molecular response; IS, international scale; McyR, major cytogenetic response; MMR, major molecular response; MR, molecular response.

**TABLE 2:** Clinical studies of TKI discontinuation.

Study	Year of publication	No. of patients	TKI used	Definition of DMR	Median duration of DMR, month	Definition of molecular relapse	TFR Rate
GIMEMA CML 0307^81^	2022	36	Nilotinib	MR4.0	74 (7–110)	Loss of MMR	24.7% at 2 years
STIM2^35^	2022	199	Imatinib	MR4.0	38.3 (22.95–124.3)	Loss of MMR	34.5% (95%CI: 25.6–43.3) at 9 years
5‐year update of the ENESTfreedom trial^70^	2021	190	Nilotinib	MR4.5	30.4 (26.2–57.9)	Loss of MMR	42.6% at 5 years
LAST^23^	2021	172	Bosutinib, Dasatinib, Imatinib, Nilotinib	MR4.0	56.6 (25.1–176.6)	Loss of MMR	65.5% at 1 year
DASFREE^71^	2020	84	Dasatinib	MR4.5	28 (13–116)	Loss of MMR	46% at 2 years
DADI^82^	2020	58	Dasatinib	BCR::ABL% < 0.0069%	23.3 (37.9–77.5)	Loss of MMR	55% at 6 months
ISAV^83^	2020	107	Imatinib	UMRD	25.8 (18–99)	Loss of MMR	48% at 36 months
Prospective Brazilian Trials^84^	2019	48	Imatinib	MR4.5	76 (23–135)	Loss of MMR	61% (95% CI: 47–75) at 20 months
NILSt^85^	2019	87	Nilotinib, Imatinib	MR4.5	NA	Loss of MMR	60.9% (90% CI: 51.6–69.7) at 3 years
DOMEST^86^	2019	110	Imatinib	MR4.0	55 (24–133)	Loss of MR4.0	64.3% at 24 months
Fava, C. et al^87^	2019	293	Bosutinib, Dasatinib, Imatinib, Nilotinib	MR4.0	46 (31–74)	Loss of MMR	62% (95% CI: 56;68) at 34 months
JALSG‐STIM213^73^	2018	68	Imatinib	MR4.0	30.6 (17.6–59.9)	Loss of MMR	67.6% at 12 months
STAT2^19^	2018	78	Nilotinib	MR4.5	47.9 (6–128)	Loss of MMR	67.9% (95% CI: 56.4–78.1) at 12 months
EURO‐SKI^74^	2018	755	Imatinib, Nilotinib, Dasatinib	MR4.0	56.4 (34.8–6·82.8)	Loss of MMR	50% (95% CI: 46–54) at 24 months
Mahon, F. X. et al^88^	2018	126	Imatinib, Nilotinib	MR4.5	19.8 (0.3–78)	Loss of MMR	53% (95% CI: 44–62) at 96 weeks
D‐STOP^89^	2018	54	Imatinib, Dasatinib	MR4.0	51 (24–173)	Loss of MMR	62.9% (95% CI: 48.5–74.2) at 12 months
Hernández‐Boluda, J. C. et al^90^	2018	236	Imatinib, Nilotinib, Dasatinib, Bosutinib, Ponatinib	MR4.5	68 (40–100)	Loss of MMR	64% (95% CI: 55–72) at 4 years
STOP 2G‐TKI^43^	2017	60	Dasatinib, Nilotinib	MR4.5	29 (24–64)	Loss of MMR	53.57% (95% CI: 40.49–66.65) at 48 months
ENESTfreedom^91^	2017	190	Nilotinib	MR4.5	18.3 (0.3–70.9)	Loss of MMR	51.6% (95% CI 44.2–58.9) at 48 weeks
STIM1^92^	2017	100	Imatinib	UMRD	N/A	Loss of MMR	38% (95% CI:29–47) at 60 months
KID^93^	2016	90	Imatinib	UMRD	39.9 (22.1–130.7)	Loss of MMR	58.5% ± 5.2% at 24 months
DADI^42^	2015	63	Dasatinib	BCR::ABL% < 0.0069%	N/A	BCR::ABL% > 0.0069%	48% (95%CI: 35–59) at 12 months
A‐STIM^94^	2014	80	Imatinib	UMRD	41 (24–96)	Loss of MMR	61% (95% CI: 51–73) at 36 months
HOVON^95^	2013	15	Imatinib	MR4.5	20 (7–60)	Loss of MR4.5	33% (95%CI: 44–88) at 24 months
TWISTER^26^	2013	40	Imatinib	UMRD	N/A	Loss of MMR	47.1% at 24 months
STIM2^96^	2013	124	Imatinib	MR4.0	N/A	Loss of MMR	61.2% at 12 months
Yhim, H. Y. et al^97^	2012	14	Imatinib	BCR::ABL% < 0.0046%	N/A	BCR::ABL% > 0.0046%	28.6% at 12 months
Keio STIM^98^	2012	40	Imatinib	BCR::ABL% < 0.0046%	N/A	BCR::ABL% > 0.0046%	55.4% at 12 months
STIM^63^	2010	100	Imatinib	MR5.0	35·5 (24·0–85·0)	Loss of MR5.0	41% at 24 months

Abbreviations: N/A, not available; MMR, major molecular response; MR, molecular response.

**TABLE 3:** Information of ongoing clinical trial.

ClinicalTrials ID	Study start	Primary completion	Study completion	Enrollment	Drug used	Primary objective
NCT01784068	2013‐03‐04	2016‐05‐31	2025‐02‐20	215	Nilotinib	48 weeks TFR rate
NCT04838041	2021‐11‐11	2028‐06	2029‐07	51	Asciminib and Imatinib	The 12‐month “second” TFR rate
NCT05439889	2022‐08‐11	2028‐08‐11	2032‐08‐11	100	N/A	TFR rate at the end of the study
NCT03817398	2019‐11‐08	2026‐06‐30	2026‐06‐30	110	N/A	2‐year TFR rate
NCT02852486	2016‐06‐22	2024‐02	2024‐02	31	Imatinib	TFR rate at the end of the study
NCT03610971	2019‐11‐19	2025‐01	2025‐01	51	Ruxolitinib and TKI	The 24‐month “second” TFR rate
NCT03874858	2019‐03‐22	2026‐07‐31	2026‐12‐31	103	Nilotinib and Asciminib	1‐year TFR rate
NCT01698905	2012‐12‐20	2015‐11‐26	2025‐02‐06	163	Nilotinib	48 weeks TFR rate
NCT05753384	2023‐10‐15	2028‐10‐15	2029‐01‐15	140	N/A	24 months TFR rate
NCT02602314	2016‐11‐11	2023‐02	2024‐02	450	Imatinib and Nilotinib	TFR rate at the end of the study
NCT04769947	2021‐08‐09	2022‐07‐08	2030‐02‐08	800	N/A	1‐year TFR rate
NCT05926128	2019‐02‐01	2022‐06‐01	2025‐01	75	N/A	TFR rate
NCT02268370	2023‐06‐19	2025‐12	2025‐12	164	Imatinib and imatinib	TFR rate
NCT05152537	2021‐01‐01	2023‐06	2023‐12	15	N/A	Correlation of FLOR3 with TFR
NCT04160546	2020‐01‐17	2024‐12‐02	2024‐12‐02	80	Ponatinib and Acetylsalicylic acid	TFR rate
NCT04147533	2020‐06‐16	2026‐06	2026‐06	150	N/A	TFR rate

Abbreviations: N/A, not available; TKI, tyrosine kinase inhibitor; TFR, treatment‐free remission.

## 2 CURRENT GUIDELINES FOR TFR

To explore TFR better, we should first understand the latest guidelines for TKI discontinuation. Both the European Leukemia Net (ELN) and the National Comprehensive Cancer Network (NCCN) include the detailed criteria for TKI discontinuation (Table 4). The 2020 ELN recommendations regarding TFR are divided into mandatory, minimal, and optimal requirements.^13^ First, the ELN guidelines require that patients with TKI discontinuation be in their first CML‐CP, actively be engaged in structured communication before implementation, and follow the conditions for performing high‐quality rapid quantitative PCR based on the International Scale (IS). The patient needs to agree with monthly monitoring for the first 6 months, followed by monitoring every 2 months from 6 to 12 months, and every 3 months thereafter. For standardized monitoring, patients need to have typical e13a2 or e14a2 BCR::ABL1 transcript, to have been on TKI for more than 5 years or on two generations of tyrosine kinase inhibitor (2GTKI) for more than 4 years, and to have achieved DMR for more than 2 years, with no prior treatment failure. TKI treatment for more than 5 years, MR4 lasting for more than 3 years, and MR4.5 lasting for more than 2 years are considered optimal conditions. The latest V3.2022 NCCN guidance for CML requires that, before TKI discontinuation, patients be over 18 years old in the CML‐CP, with no prior history of accelerated or blast phase CML, and have quantifiable BCR::ABL1 transcripts.^14^ For the monitoring method, the NCCN guidelines have more detailed criteria, requiring a qPCR assay with a sensitivity of at least MR4.5 and results to be available within 2 weeks. The NCCN guidelines require patients to have had MR4 for more than 2 years (at least four tests with at least 3 months between each test) before stopping TKI. The frequency of monitoring after discontinuation is similar to that mentioned in the 2020 ELN guidelines; however, patients who remain in the MMR would require quarterly monitoring after the first year. The NCCN also provides advice after treatment discontinuation. TKI therapy would be resumed within 4 weeks of MMR disappearance, whereas molecular monitoring would be performed monthly until MMR recovery. Monitoring every 3 months thereafter is recommended indefinitely for patients who have reinitiated TKI therapy after MMR loss. For patients who have not recovered their MMR after 3 months, testing for BCR::ABL1 kinase domain mutations should be performed, followed by monthly molecular monitoring over the next 6 months.

**TABLE 4:** Criteria for TKI discontinuation.

Criteria	ELN 2020	NCCN 2022 V3
Age	N/A	≥18
Medical history	CML in first CP only No prior treatment failure (expect TKI intolerance)	CML in first CP only
BCR::ABL transcript	Typical (e13a2 or e14a2)	Quantifiable
Duration of TKI therapy	>5 years >4 years for 2GTKI	≥3 years
Molecular response	MR4 > 2 years	MR4 ≥ 2 years (documented on at least four tests, performed at least 3 months apart)
Monitoring method	High‐quality qPCR using the IS with rapid turnaround of PCR test results	Reliable qPCR with a sensitivity of detection of at least MR4.5 provides results within 2 weeks
Frequency of monitoring	Monthly in the first 6 months, bimonthly during months 7–12, and quarterly thereafter	Monthly in the first 6 months, bimonthly during months 7–12, and quarterly thereafter
Loss of MMR	N/A	Prompt resumption of TKI within 4 weeks BCR::ABL1 kinase domain mutation testing for those who fail to achieve MMR after 3 months

Abbreviations: 2GTKI, 2 generation tyrosine kinase inhibitor; CP, chronic phase; MMR, major molecular response; MR, molecular response; N/A, not available.

## 3 MONITORING BEFORE TKI DISCONTINUATION

### 3.1 Depth of molecular response

TFR should be attempted only if the condition of the patient with CML is well controlled. Currently, qRT‐PCR is used internationally as the gold standard for detecting BCR::ABL transcripts,^15^ and its detection results are used as important indicators for evaluating the efficacy and prognosis of patients with CML. Digital droplet PCR (ddPCR), which has emerged recently, uses a water–oil emulsion droplet system that divides the sample into thousands of individually repeated PCRs.^16^ This high repeatability significantly improves the sensitivity of detection and is not affected by amplification differences.

Although ddPCR technology has not been widely promoted internationally, it has been used to explore TKI discontinuation owing to its high sensitivity in detecting BCR::ABL1 transcripts.^11^, ^17^, ^18^ Several high‐quality studies using qRT‐PCR have demonstrated the importance of monitoring minimal residual disease (MRD) during TKI therapy. A multicenter, phase II, single‐treatment arm, open‐label clinical trial, conducted by Takahashi et al., enrolled 78 patients with CML‐CP.^19^ They monitored patient MRD using qRT‐PCR with an accuracy of no less than MR4.5. The results showed that the treatment‐free survival curve was significantly better in patients without undetectable MRD (Undetectable MRD was defined as undetectable BCR::ABL1 transcript with MR5) than in patients with undetectable MRD (3‐year treatment‐free survival, 75.6% vs. 48.6%, respectively; *p* = 0.0126 by the log‐rank test).^19^ A single‐center, prospective, pilot cohort clinical trial conducted by Seguro et al. enrolled 31 patients with CML‐CP.^20^ All participants were monitored in the same laboratory since diagnosis using qRT‐PCR, with a sensitivity of at least MR5. The results showed that a more profound molecular response (MR4.5) was associated with a lower risk of molecular relapse.^20^ In the TKI discontinuation trial, qRT‐PCR with a sensitivity of at least 0.01% (4 log reduction or MR4) on the International Scale (IS) was the standard definition of DMR.^21^ However, owing to the differences in the sensitivity of qRT‐PCR in different laboratories, some clinical trials cannot guarantee that all monitored samples come from the same laboratory, which leads to uncertainty regarding whether a patient at MR4 is actually at MR4.5 or MR5. Owing to its high sensitivity and accuracy, ddPCR can overcome these limitations. Studies comparing qRT‐PCR and ddPCR monitoring have shown that ddPCR highlights the high heterogeneity of MRD levels in patients belonging to the same MR class before TKI discontinuation.^11^ Moreover, absolute numbers of BCR‐ABL1 copies/μL were detected and measured by ddPCR in MR4.5 or MR5 patients with undetectable BCR‐ABL1 transcripts on qRT‐PCR.^11^ Presently, ddPCR is effective for exploring the field of TFR. According to the conversion coefficients of ddPCR and BCR::ABL1 ratios stipulated by IS, the significant cutoff point is usually set to 0.0023%.^22^ The TFR study by Nicolini et al. used ddPCR as a means of molecular monitoring and showed that MRD, as determined by ddPCR, is a key factor for predicting successful treatment‐free remission in patients with CML‐CP.^22^ Similarly, the results of a nonrandomized clinical trial showed that the molecular recurrence rate of patients without UMRD by both ddPCR and qRT‐PCR was 10.3% (nine of 87), which was much lower than that of patients without UMRD detected by qRT‐PCR (64.3%, 36 of 56).^23^ Although there are some limitations to qRT‐PCR, there is consistency between qRT‐PCR and ddPCR regarding the effect of the depth of molecular response on TFR acquisition. More targeted and personalized TKI therapy has increased the need for a more sensitive quantitative analysis of MRD, and more high‐quality clinical trials would be required to further optimize ddPCR applications, so that ddPCR can be widely used for routine MRD monitoring in patients with CML.

### 3.2 BCR::ABL1 DNA

DNA‐PCR for BCR::ABL1 has several advantages over RNA‐PCR. First, DNA samples are more stable than RNA samples, thereby reducing degradation during sample transport. Second, DNA‐PCR standardization can use upstream control genes rather than cell numbers.^24^ However, DNA‐PCR is difficult to implement compared to RNA. Owing to the large breakpoint region between BCR and ABL in the BCR::ABL1 fusion gene, multiple primers are required to determine the specific breakpoint. A study of BCR::ABL1 genomic DNA‐PCR response kinetics showed that BCR::ABL1 DNA could be detected in 48% of samples in which BCR::ABL1 mRNA could not be detected.^25^

Recently, there have been clinical studies incorporating BCR::ABL1 DNA‐PCR.^1^, ^26^, ^27^ Ross et al. conducted a prospective trial in 40 patients with CML‐CP and showed a higher incidence of TFR in patients with undetectable BCR::ABL1 DNA; however, the difference was not statistically significant.^26^ A recent study proposed a “traffic light” model based on DNA and RNA analysis during DMR maintenance prior to TKI discontinuation. According to the detection of BCR::ABL1 DNA and RNA, the model was divided into three levels of green, yellow, and red.^27^ Green indicated no DNA or RNA residue detected by ddPCR, indicating a low recurrence rate (20% relapse after 18 months). Yellow indicated that ddPCR only detected DNA and not RNA, suggesting a moderate recurrence rate (43% relapse after 18 months). Red indicated that ddPCR detected residual BCR::ABL1 DNA as well as RNA before TKI discontinuation, implying that the patients had a higher recurrence rate after discontinuation (80% relapse after 18 months).^27^ This traffic light stratification model provided new possibilities of prediction before TKI discontinuation. A more precise study by Pagani et al. reported similar results.^1^ Sample cells were sorted into granulocytes, monocytes, B lymphocytes, T lymphocytes, and NK cells before DNA‐PCR. Of the 20 patients who maintained TFR for more than 1 year, residual BCR::ABL1 DNA was detected in 18, all in the B lymphocyte population; in a small number of patients, it was also detected in monocytes, T lymphocytes, and NK cells. Notably, BCR::ABL DNA residues were not found in granulocytes of the 20 patients, although they were found in the granulocytes of all patients who relapsed within 3 months after TKI discontinuation.^1^ Thus, the detection of BCR::ABL1 DNA in granulocytes seemed to predict molecular relapse during TFR. However, further studies would be required to confirm this conclusion. Moreover, whether cell sorting can be broadly performed in all laboratories would need further consideration.

### 3.3 Type of BCR::ABL1 transcripts

**FIGURE 1:**
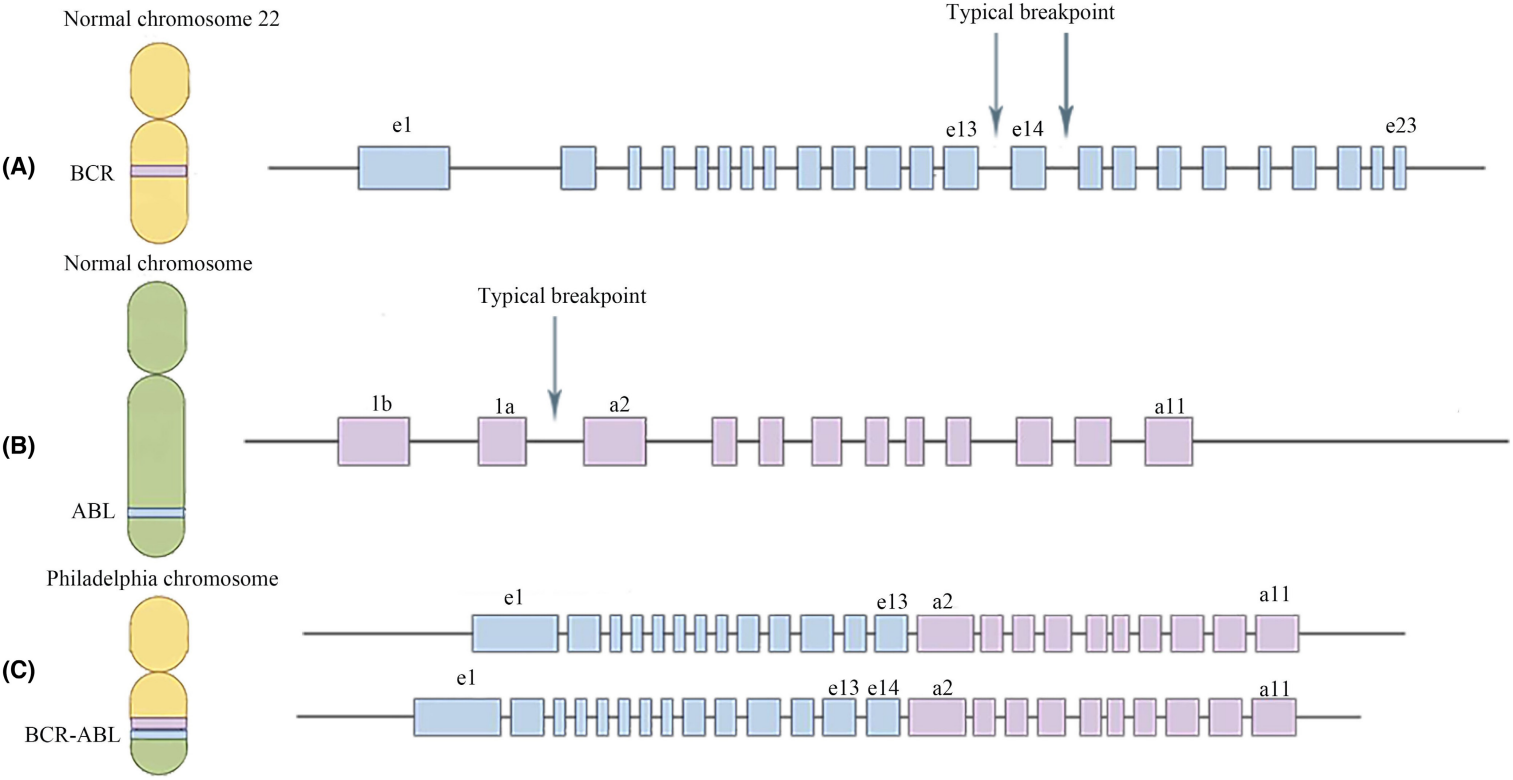
Typical BCR::ABL1 transcripts. (A) The approximate location of the Typical breakpoint of the BCR gene on chromosome 22. (B) The approximate location of the Typical breakpoint of the ABL gene on chromosome 9. (C) Two kinds of typical transcripts of the BCR::ABL1 fusion gene on the Philadelphia chromosome.

CML originates from a translocation in chromosomes 9 and 22. In most patients, the breakpoints on chromosome 9 are located upstream of exon 2 of the ABL1 gene, whereas those on chromosome 22 are located downstream of exon 13 or 14 of the BCR gene (Figure 1). The fusion genes produced at these breakpoints are referred to as e13a2 and e14a2, which also called major BCR::ABL1 or p210. The proportion of patients with e13a2 and/or e14a2 is approximately 98%.^28^

Transcription type is considered a variable that may influence treatment response and TFR.

e14a2 has been suggested to have a relatively better therapeutic outcome and prognosis than e13a2.^29^, ^30^ However, other studies have found no significant difference in long‐term survival between patients expressing e13a2 and e14a2.^31^, ^32^

A similar situation was observed with respect to whether the transcription type affected TFR; some studies found the expression of e14a2 to be associated with a higher rate of TFR,^33^, ^34^ although the results of other studies showed no significant relationship between transcription type and TFR.^20^, ^35^

Recent studies have suggested that at least some of these differences between patients with e13a2 and e14a2 transcript types can be explained by technical factors, in addition to the different potential biological roles of the oncoproteins that each translates.^36^, ^37^, ^38^ Since the e13a2 transcript is reduced by 75 bp compared to the e14a2 transcript, qRT‐PCR had a higher amplification efficiency for e13a2 than e14a2. This led us to overestimate the e13a2 BCR::ABL1 residue.^36^ This could partly explain why patients expressing e13a2 achieve DMR and TFR more quickly and easily, respectively. Notably, ddPCR effectively eliminates this bias. We found that an experiment using ddPCR, to detect BCR::ABL1 residues, did not reveal a significant effect of transcription type on the TFR.^35^ There could be a 1.8–6.5‐fold difference between the different transcripts^36^; therefore, we recommended using ddPCR for exploring the effect of transcription type on TFR.

Since standard qRT‐PCR is inefficient in detecting atypical fusion genes and is not suitable for molecular monitoring, patients often require customized PCR protocols or FISH analysis.^39^ Most guidelines do not support attempts to discontinue treatment for patients with atypical transcripts. However, a TKI discontinuation trial by Dragani et al. in patients expressing atypical transcripts showed promising results, with five of seven patients maintaining a TFR for more than 19 months.^40^ This suggested that under strict monitoring, TKI discontinuation may be attempted in patients with CML who are expressing atypical transcripts.

There is currently no guideline to recommend transcript types for TFR, and there are not enough clinical trials to support the significant effects of different transcript types on TFR; therefore, we cannot recommend that transcript types be considered before considering TKI discontinuation (only e13a2 and e14a2).

### 3.4 BCR::ABL1 kinase domain mutations

Mutations in the BCR::ABL1 kinase domain (KD) are considered to be the main cause of acquired TKI resistance^13^, ^14^ (Table 5, ^41^). TKI resistance affects the efficacy of CML therapy, and has been found to be an adverse factor in TFR maintenance in some TKI discontinuation trials.^42^, ^43^, ^44^ Studies have found the median time for BCR::ABL1 doubling in imatinib‐resistant patients to be significantly shorter than that in nonresistant patients,^42^, ^44^ which might explain why imatinib‐resistant patients are more prone to molecular relapse after TKI discontinuation. The latest NCCN guidelines for TKI discontinuation no longer exclude patients with TKI resistance.^14^ In a recent TKI discontinuation trial on 10 patients with a history of BCR::ABL1 kinase domain mutations, five gained TFR for more than a year.^45^ This suggested that patients with TKI resistance may indeed achieve a TFR. Sanger sequencing is the most widely used technique to detect KD mutations, with a sensitivity of approximately 10%–20% at BCR::ABL1 ratio ≥1%.^46^ Therefore, if Sanger sequencing is used to detect KD mutations in patients in TKI discontinuation trials, it must be performed in the treatment phase before TKI discontinuation.^47^ The NGS‐based *BCR‐ABL1* KD mutation screening conducted by Soverini et al showed evidence of mutations undetectable by Sanger sequencing in 80 out of 236 patients (34%), of whom 42 (18% of the total patient population) had low‐level mutations somehow relevant for clinical decision‐making,^48^ which means the sensitivity and accuracy of NGS for KD mutation detection were significantly better than Sanger sequencing. However, if we choose ddPCR and next‐generation sequencing with higher sensitivity (approximately 0.2% detection limit), a new problem might arise; the significance of low‐level mutations detected by this high‐sensitivity technology is not yet clear in case of patients without TKI resistance and it cannot, therefore, be a predictor of TFR. However, KD mutations are not present in all patients who develop TKI resistance, and some resistance mechanisms still remain unknown. Whether patients with BCR::ABL1 KD mutations can maintain long‐term stable TFR remains to be confirmed in larger clinical trials.

**TABLE 5:** Activity of TKIs against kinase domain mutations of BCR::ABL1.

Mutations	Imatinib	Bosutinib	Dasatinib	Nilotinib	Ponatinib
M244V	++++	++++	++++	++++	+++
L248R	+	+	+	+	++
L248V	+++	+++	++	+++	+++
G250E	++	++	++	++	++
Q252H	++++	++++	+++	+++	++
Y253F	+++	++++	++++	+++	+++
Y253H	++	++++	+++	+	+++
E255K	++	++	++	++	++
E255V	+	++	+++	+	+
D276G	+++	++++	++++	++++	+++
E279K	+++	++++	++++	++++	+++
E292L	++++	++++	++++	++++	++++
V299L	++++	+	++	++++	++++
T315A	++++	++	+	+++	++++
T315I	+	+	+	+	+++
T315V	+	+	+	+	+++
F317L	+++	+++	++	+++	++++
F317R	+++	+	+	+++	++
F317V	++++	+	+	++++	+++
M343T	++++	++++	++++	++++	++++
M351T	++++	++++	++++	++++	++++
F359I	++	+++	+++	+	+++
F359V	+++	++++	++++	++	++
L384M	++++	++++	+++	+++	+++
H396P	+++	++++	++++	+++	++++
H396R	+++	++++	++++	+++	++
F486S	++	+++	+++	++++	+++

*Note*: +, highly resistant; ++, resistant; +++, moderately resistant; ++++, sensitive.

### 3.5 CML stem cells

BCR::ABL1 in patients with CML originates from a cell with intrinsic or acquired biological potential to cause leukemia, such as a CML stem cell (LSC).^49^, ^50^ Although traditional TKI therapy has a strong antiproliferative effect on LSC, it has a poor ability to induce apoptosis,^51^ especially in quiescent LSCs. One study reported that some signaling pathways do not depend on BCR::ABL1 to maintain the survival of LSC in the quiescent stage,^52^ which makes TKI treatment ineffective. Although the ideal situation for attempting TFR is to clear the LSC from the body, there is currently no treatment specific to LSCs in the clinic. However, several clinical trials have found residual CML LSCs in most patients who maintain TFR, even when BCR::ABL is monitored using a highly sensitive assay, such as ddPCR.^53^, ^54^ The data suggested that LSCs are present even in patients who achieve DMR, and that TFR does not appear to require complete eradication of LSCs in at least a subset of patients. However, it should be noted that a considerable number of patients are still unable to achieve TFR. Since very little is known about LSCs till date and there is no specific clinical therapy for LSCs, current attempts to treat TFR do not consider the impact of LSCs. There have been studies on LSC‐specific therapy,^55^, ^56^ and we believe that these therapies aimed at LSCs can, in future, effectively reduce molecular recurrence after TKI discontinuation.

In recent years, the development of single‐cell sequencing has led to the discovery of specific gene expression patterns in patients with poor response to TKI therapy, including some LSC phenotypes.^57^ For example, during treatment, Lin^−^CD34^+^CD38^−/low^CD45RA^−^cKIT^−^CD26^+^ LSCs showed increased expression of genes related to proliferation and decreased expression of genes related to quiescence.^58^ In future, we may screen patients, who are not suitable for TFR, based on the expression pedigree of LSCs.^59^

## 4 MONITORING AFTER TKI DISCONTINUATION

Rigorous molecular monitoring is necessary after the formal discontinuation of TKIs. Since the molecular depth inclusion criteria for TFR trials are usually MR4 or MR4.5, we recommend that laboratories or hospitals conducting TKI withdrawal tests perform qRT‐PCR or ddPCR with a sensitivity of at least MR4.5. In patients aiming at TFR, molecular relapse tends to occur within the first 6 months after TKI discontinuation.^60^ Therefore, adequate molecular monitoring with sufficient frequency and sensitivity should be ensured during the first year after discontinuation to prevent delays in the optimal treatment opportunity for patients with molecular relapse. However, excessive monitoring programs may reduce the patients' willingness to try TFR and impose additional financial burdens, especially for those with poor financial conditions and in areas where monitoring is expensive. Past TKI discontinuation trials tended to monitor monthly for the first 12 months^61^; however, a model of the safe minimum frequency for molecular monitoring in patients with CML attempting TFR by Ross et al. suggested that this frequency may not be necessary.^62^ The model suggested that monthly testing for the first 6 months and alternate‐month testing during 6–12 months would not delay the resumption of TKI therapy in relapsed patients while reducing the number of tests by nearly one‐third.^62^ A previous study had shown that the median time for BCR::ABL1 to double in patients with molecular recurrence is approximately 1‐log per month.^63^ Therefore, if a patient begins to discontinue TKI at a stable MR4.5, and BCR::ABL1 increases at the above rate, it will take at least 2 months for patients with molecular relapse to lose their MMR. Therefore, monthly molecular monitoring during the first year after TKI discontinuation was not necessary. However, it would be worth noting that the frequency of monitoring is not static. Six months after TKI discontinuation, if the patient tends to lose MMR, the frequency of monitoring should be restored to once a month to prevent any delay in treatment. It would further be worth noting that the time required to obtain the test sample and test results is equally important. The ideal time is to obtain results within 2 weeks and act on them quickly.^14^ If the patient loses MMR, TKI therapy is immediately resumed along with monthly molecular monitoring. In conjunction with the current guidelines and clinical trials described above,^13^, ^14^, ^62^ we recommend monthly molecular monitoring for the first 6 months after TKI discontinuation, bimonthly monitoring during 6–12 months, and quarterly monitoring thereafter. If a patient tends to lose MMR (e.g., the patient has lost DMR, and the most recent test result is close to MR3), monthly monitoring is recommended, regardless of the duration of discontinuation. In addition, since the international standard molecular monitoring protocol is for patients with typical CML (transcripts e13a2 and/or e14a2), the above recommendations are only applicable to this subset of patients. Patients with rare transcripts would require a relatively individualized monitoring regimen.

## 5 SECOND ATTEMPT TO TFR

If a patient with CML, who attempts to stop TKI therapy, has a molecular relapse, the possibility of achieving TFR in such a case may be a concern. Fortunately, the vast majority of patients in whom TFR attempts have failed can quickly regain DMR after resuming TKI therapy, creating conditions for these patients to attempt TFR for a second time. A small‐scale study on 16 patients who underwent a second attempt of TFR revealed different recurrence dynamics after the second withdrawal of TKI. Five patients had a slower second recurrence than the first, whereas five others had a faster second relapse.^64^ Till date, the largest study on the second attempt of TFR was RE‐STIM in France, which included 70 patients who had a molecular relapse after the first TKI discontinuation, regained DMR, and stopped the TKI again.^65^ In the end, 35% of patients in the trial maintained TFR after 36 months of follow‐up, and the depth of BCR::ABL1 molecular response at 3 months after the first TFR attempt was a factor in the second TFR attempt; that is, patients who retained MR4.5 at 3 months after the first TKI discontinuation had a higher success rate (46%) in the second TFR attempt. However, the criterion for the first restoration of TKI therapy in this trial was loss of MR4.5, whereas that for the second restoration of TKI therapy was loss of MMR. Another trial involving a second TFR attempt reported that six out of 12 patients remained in MMR (median follow‐up, 8.6 years), and the most recent recurrence occurred 6 years after the second TKI discontinuation.^66^, ^67^ Reasons underlying the successful discontinuation, after the first TKI discontinuation failure, still remain unclear; however, one possible explanation could be the progressive exhaustion of quiescent CML stem cells.^68^ Currently, very few clinical trials have been conducted on the second TFR attempts. A meta‐analysis published in 2020 reported that only 124 patients underwent a second attempt of TFR before publication.^69^ Although such limited data are insufficient to assist in formulating clinical standards for the second TFR, attempt for the latter can be suggested to be safe and feasible. Dulucq et al. estimated that the molecular relapse rates of patients after the first discontinuation at 0–6, 6–12, and 12–18 months were 35%, 8%, and 3%, respectively, based on the previous TFR clinical trials. The molecular recurrence rates after the second discontinuation were 48%, 27%, and 12%, respectively.^69^ Although there was no significant overall difference in molecular relapse rates between the first and second discontinuations, the time point of molecular relapse after the second discontinuation was more dispersed. Therefore, the molecular monitoring strategy for the second TFR attempt should be appropriately adjusted. We recommend molecular monitoring once a month for the first 12 months, every alternate month for 12–18 months, and every quarter during the second attempt to TFR.

## 6 ISSUES STILL TO BE CONSIDERED

Although the abovementioned detection methods for BCR::ABL1 can guide the progression of TFR to a certain extent, it is often impossible or difficult to perform the detection techniques, such as ddPCR and DNAPCR, in conventional clinical setting. This involves not only the technical facilities of the local hospital but also the economic situation of the patients and cost‐effectiveness of the treatment. Consequently, many highly sensitive detection methods exist only in clinical trials, but cannot be used for conventional treatments. Here we summarize the advantages and disadvantages of these monitoring methods again (Table 6).

**TABLE 6:** Summary of advantages and disadvantages of monitoring methods.

	Advantages	Disadvantages
qRT‐PCR	Sensitive, widely used, as the gold standard for detecting BCR::ABL transcripts	Affected by sequence length and technical conditions
ddPCR	More accurate, sensitive, and robust than qRT‐PCR	Difficult to use on a large scale
NGS	Great sensitivity and accuracy for kinase domain mutations	Expensive, and the significance of some low‐level mutations detected is unclear

Abbreviations: ddPCR, digital droplet PCR; NGS, next‐generation sequencing; qRT‐PCR, quantitative reverse transcription‐PCR.

Many clinical trials on the withdrawal of TKI have found that longer the duration of TKI treatment and DMR, higher the success rate of TFR.^22^, ^23^, ^35^, ^70^, ^71^, ^72^, ^73^, ^74^ In fact, this is an expected outcome; longer the treatment time and duration of DMR maintenance, better is the prognosis, and easier it is to attempt TFR. However, according to this conclusion, there will never be the best time for discontinuing TKI, since it is always better to stop TKI therapy later. An optimal time is required to stop taking the drugs. Some scholars have conducted preliminary research and recommended 6 years as the shortest duration of imatinib use, based on positive and negative predictive values.^75^

Some patients, who underwent TFR, experienced withdrawal symptoms after stopping TKI therapy, including muscle and bone pain.^76^, ^77^, ^78^ The symptoms usually lasted over months, beginning within days or weeks of drug discontinuation. However, the mechanisms underlying TKI withdrawal syndrome remain unclear. Possible causes include c‐Kit inhibition, bone remodeling, and mast cell activation.^79^ Although a small number of patients require medications, such as cortisol,^80^ most withdrawal symptoms are mild and self‐limiting. Since patients who are aiming for TFR are often in a state of anxiety due to the fear of relapse, the withdrawal symptoms need to be explained to them in detail before stopping TKI, thereby reducing unnecessary anxiety.

## 7 CONCLUSIONS

**FIGURE 2:**
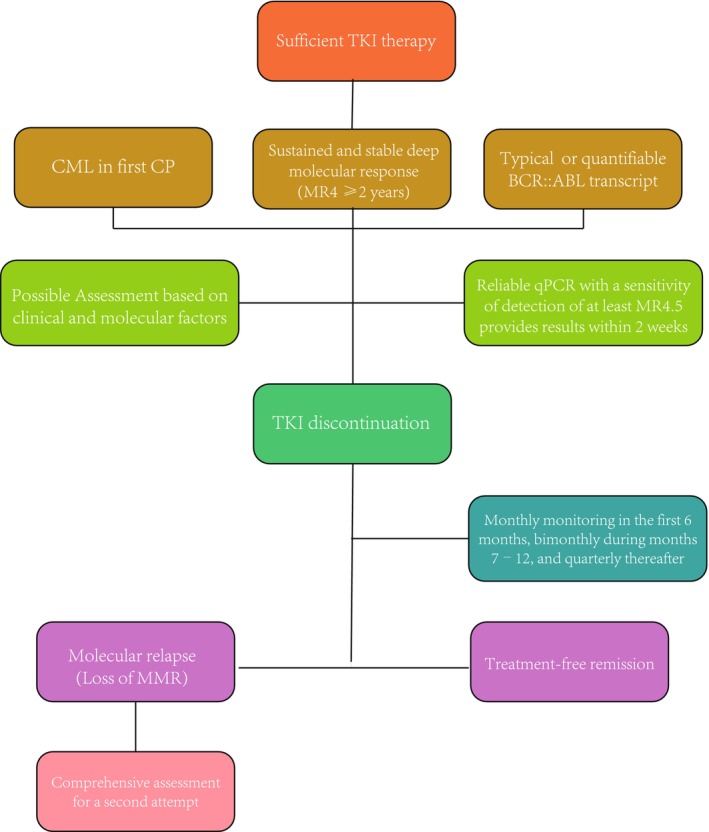
Approach to treatment‐free remission. Combining clinical indicators with molecular markers and using precise and efficient molecular monitoring to achieve treatment‐free remission is an ideal treatment model and it is also the focus of future research in the field of CML.

Based on the current clinical guidelines and the factors influencing the TFR obtained in some clinical trials, TKI discontinuation therapy has been initiated in some patients with DMR. Herein, we discussed some of the factors that may be related to TFR outcomes at the molecular level, along with some monitoring strategies. However, till date, most of these factors have not been involved in decision‐making for clinical TFR. Currently, predictive indicators for TFR outcomes and recurrence are lacking in clinical practice. In future, TFR research should focus on combining the clinical indicators with molecular monitoring and biological markers to personalize patient conditions and guide clinicians to develop individualized treatment plans, so that more patients with CML can achieve safer and stabler TFR (Figure 2).

## AUTHOR CONTRIBUTIONS

**Zhao Zhang:** Data curation (equal); formal analysis (equal); investigation (equal); methodology (equal); resources (equal); visualization (equal); writing – original draft (equal); writing – review and editing (equal). **Xianghui Zhou:** Data curation (equal); formal analysis (equal); investigation (equal); methodology (equal); resources (equal); visualization (equal); writing – original draft (equal); writing – review and editing (equal). **Xin Zhou:** Data curation (equal); formal analysis (equal); investigation (equal); methodology (equal); resources (equal); visualization (equal); writing – original draft (equal); writing – review and editing (equal). **Zhipeng Cheng:** Conceptualization (equal); funding acquisition (equal); project administration (equal); supervision (equal); validation (equal); writing – review and editing (equal). **Yu Hu:** Conceptualization (equal); funding acquisition (equal); project administration (equal); supervision (equal); validation (equal); writing – review and editing (equal).

## FUNDING INFORMATION

Supported by Grants from the National Key R&D Program of China (No.2022YFC2304600) and National Natural Science Foundation of China (No.81800134).

## CONFLICT OF INTEREST STATEMENT

The authors have no conflict of interest.

## Data Availability

The authors declare that all data supporting the findings of this study are available within the article or its supplementary information files.
